# Relationship between psychodynamic functioning, defensive mechanisms, and trauma in patients with post-traumatic stress disorder (PTSD)

**DOI:** 10.47626/2237-6089-2022-0546

**Published:** 2024-11-26

**Authors:** Taís Cristina Favaretto, Luciane Maria Both, Silvia Pereira da Cruz Benetti, Lúcia Helena Machado Freitas

**Affiliations:** 1 Universidade Federal do Rio Grande do Sul Programa de Pós-Graduação em Psiquiatria e Ciências do Comportamento Porto Alegre RS Brazil Programa de Pós-Graduação em Psiquiatria e Ciências do Comportamento, Universidade Federal do Rio Grande do Sul (UFRGS), Porto Alegre, RS, Brazil.; 2 Universidade do Vale do Rio dos Sinos São Leopoldo RS Brazil Universidade do Vale do Rio dos Sinos (UNISINOS), São Leopoldo, RS, Brazil.; 3 UFRGS Departamento de Psiquiatria e Medicina Legal Porto Alegre RS Brazil Departamento de Psiquiatria e Medicina Legal, UFRGS, Porto Alegre, RS, Brazil.; 4 Hospital de Clínicas de Porto Alegre Serviço de Psiquiatria Porto Alegre RS Brazil Serviço de Psiquiatria, Hospital de Clínicas de Porto Alegre (HCPA), Porto Alegre, RS, Brazil.; 5 Núcleo de Estudos e Tratamento do Trauma Psíquico Serviço de Psiquiatria Porto Alegre RS Brazil Núcleo de Estudos e Tratamento do Trauma Psíquico, Serviço de Psiquiatria, HCPA, Porto Alegre, RS, Brazil.

**Keywords:** Violence, psychological trauma, post-traumatic stress disorder, psychoanalytic theory, defensive mechanisms

## Abstract

**Objectives:**

Patients with post-traumatic stress disorder (PTSD) present a variety of symptoms, with different intensities, causing impairment in individual, social, and occupational functioning. The aim of this study was to understand the psychodynamic functioning of patients with PTSD, exploring relationships between symptom severity, quality of life, subjective suffering, conflicts, and psychic structure and sociodemographic characteristics, styles, and defensive mechanisms.

**Methods:**

This is a cross-sectional quantitative study with 60 participants. The following instruments were used: a sociodemographic questionnaire, the Operationalized Psychodynamic Diagnosis-2 (OPD-2), and the Defensive Style Questionnaire (DSQ-40).

**Results:**

Participants had moderate to high symptom severity, with significant subjective suffering and isolation. The main conflict was need for care vs. self-sufficiency and the level of total structure was moderate/low. Use of immature, neurotic, and mature defensive styles was observed. More primitive personality structures, more rigid defenses, and greater dependence were found in patients with history of past trauma. Other mental disorders were also associated.

**Conclusion:**

The OPD-2 was effective for assessment of the psychodynamic functioning characteristics of patients with PTSD. Therapeutic treatment should focus on the psychic structure and not only on symptom control. Prevention strategies should target vulnerability factors while strengthening protective factors.

## Introduction

Post-traumatic stress disorder (PTSD) generates emotional reactions of intense fear, anxiety, or despair.^1^ Its symptoms cause dysfunctions in patients’ global functioning and difficulties in interpersonal, social, and work relationships, leading to low quality of life.^2^ In addition, the disorder's high rate of chronicity^3^ and the large number of associated physical and mental comorbidities can culminate in suicide.^1,2^

Studies have identified sociodemographic factors,^4^ genetic and biological predispositions,^5^ history of past trauma, frequency and intensity of exposure, and other associated mental disorders, in addition to low social support, as involved in the development of PTSD.^6^ However, even in subjects diagnosed with the disorder, important differences are observed in modulation of the response to emotional regulation,^7^ development of affective personality disorders,^8^ persistence and severity of symptoms, and social behavior difficulties.^9^ Thus, different psychic functioning characteristics can confer vulnerability or protection in relation to stress processing capacities and adaptive flexibility of the psychic apparatus in the face of trauma.^10,11^

It has been identified that low level of structural personality functioning is related to the severity of post-traumatic symptoms,^11^ that accumulation of traumatic experiences is associated with the development of personality disorders,^12^ and that such disorders negatively impact the treatment of patients with PTSD.^13^ Likewise, it has been observed that patients use different defensive styles belonging to mature, neurotic, and immature factors to protect themselves from the internal perception of painful affective states.^14^ Defensive mechanisms (DM) belonging to the immature factor prevailed in war veterans^15^ and Korean refugees,^16^ while mature and neurotic factors prevail in victims of emotional abuse.^17^ Patients with depressive disorders associated with PTSD are more likely to use immature factor defensive styles than patients with anxiety disorders.^18^ However, in many cases, use of these defense mechanisms is not effective, resulting in persistence of symptoms, social withdrawal and, consequently, low quality of life.^14^

In this scenario, in a complex disorder like PTSD, a broad and deep evaluation of vulnerabilities and individual and interpersonal skills is recommended for treatment planning and intervention, seeking increased treatment adherence, relief from symptoms, and better global well-being status.^19^ The Operationalized Psychodynamic Diagnosis (OPD-2) stands out in this context as a multiaxial system that, in addition to nosological diagnosis, also yields understanding of patients’ psychodynamic functioning through a survey of their subjective suffering, resources and vulnerabilities, patterns of relational interaction, conflicts, and personality structure.^10^ The instrument can be used to assess victims of domestic violence^20^ and patients with generalized anxiety disorder (GAD),^21^ severe mental disorder,^22^ acute stress,^23^ and others.

Therefore, this study seeks to understand the psychodynamic functioning of patients with PTSD using the OPD-2. It will also explore the relationships between symptom severity, quality of life, subjective suffering, conflicts, and psychic structure and patients’ sociodemographic characteristics and their defensive styles and mechanisms.

## Method

### Participants

This is a cross-sectional quantitative study with 60 participants. Participants were selected from among people over the age of 18 who, after a traumatic event, sought care at a specialized center for study and care of trauma victims at a public service in southern Brazil and were diagnosed with PTSD. Patients were diagnosed by a psychiatrist using the Clinician-Administered PTSD Scale for Diagnostic and Statistical Manual of Mental Disorders, 5th edition (DSM-5) (CAPS-5). Sample calculation was based on a validation study carried out by Krieger^24^ presenting the Brazilian version of the OPD-2, resulting in a minimum of 53 participants. Data were collected between March 2019 and December 2020 by the researcher herself in meetings lasting an average of 1 hour and 30 minutes. During this period, 10 patients did not meet the diagnostic criteria. Another three patients were invited but declined participation and one dropped out.

### Instruments

#### Sociodemographic questionnaire:

A semi-structured questionnaire used to collect sociodemographic and clinical data.

#### OPD-2^10^

A semi-structured clinical interview used to formulate a multiaxial psychodynamic diagnosis along five axes and for planning therapy and therapeutic focus. It comprises: Axis I – Experience of the disease and prerequisites for treatment, in which the patient's general functioning, quality of life, and subjective suffering are evaluated; Axis II – Interpersonal Relationships, which integrates four interpersonal positions indicating patterns of interaction and response to objects: a) how the patient experiences himself/herself (describes his/her relational experiences and behavior); b) how the patient experiences others (identifying how he/she perceives and feels the behavior of others); c) how others (taking the interviewer into account) experience the patient, his/her offer of relationship and mode of behavior; and d) how others experience themselves in relation to the patient, their reactions, impulses and feelings (themes and items are described in Supplementary Table S1); Axis III – Conflict, which identifies the two most important conflicts in the diagnosis from among the following seven types: individuation vs. dependency, submission vs. control, need for care vs. self-sufficiency, self-worth conflict, guilt conflict, oedipal conflict, and identity conflict; Axis IV – Structure, which is composed of eight functions: self-perception, object perception, self-regulation, regulation of object relationship, internal communication, communication with the external world, attachment to internal objects, and attachment to external objects, comparing the total personality structure. Coding employs criteria with the following scores: 0 – absent, 1 – mild/not significant, 2 – moderate, 3 – high/significant, 4 – very severe/very significant, and 9 – not evaluable. For axis II, the three most prevalent items of the 32 patterns of dysfunctional relationships, themes, and resources, in each interpersonal position are scored; and Axis V- Mental and psychosomatic disorders, nosological diagnosis. In Brazil/Portugal, agreement was 78% on axis IV, 66% on axis I, and 57.7% on axis III, while axis II had a qualitative assessment based on the three items most scored by evaluators in each interpersonal position.^24,25^

#### Defensive Style Questionnaire (DSQ-40)^26^

A questionnaire with 40 statements corresponding to 20 defense mechanisms: mature factor (sublimation, humor, anticipation, and suppression), neurotic factor (pseudo-altruism, idealization, reaction formation, and undoing), and immature factor (acting out, isolation, devaluation, autistic fantasy, denial, displacement, dissociation, splitting, rationalization, somatization, projection, and passive aggression). It has a Likert response scale from 1 to 9, on which 1 indicates "strongly disagrees," 5 indicates "[patient] neither agrees nor disagrees with the statement," and 9 indicates "strongly agrees." Defense and defensive styles scores are calculated by averaging statements and defenses, respectively. The higher the value, the greater the use of defense styles. This instrument has been validated for Brazilian Portuguese, showing reliability rates assessed with Cronbach's alpha coefficient of 0.77 for the immature factor, 0.68 for the mature factor, and 0.71 for the neurotic factor.

### Data analysis

OPD-2 clinical interviews were coded by two independent judges with specific training. Kappa coefficients were independently calculated for each interview for axes I, III, IV, and V. In this study, the agreement between judges was substantial; 73% on Axis I, 76% on Axis III, 84% on Axis IV, and 100% on Axis V. For Axis II, the highest scoring relationship themes were selected and grouped. Analyses were performed using SPSS software version 25.0. Categorical variables were expressed as absolute and relative frequencies. Quantitative variables were expressed as mean ± standard deviation (mean ± SD) or median and minimum and maximum values (median [min; max]), depending on the distribution identified with the Shapiro-Wilk normality test. For comparisons in which one of the groups had fewer than 12 participants (n), quantitative variables were directly expressed as median (min; max) and compared with nonparametric Mann-Whitney or Kruskal-Wallis tests. The chi-square test was used to test for associations between qualitative variables. A cutoff of p < 0.05 was adopted for statistical significance. When significant, local association was signaled by the standardized adjusted residual analysis (values greater than 1.96).^27^

### Ethics statement

The study was approved by the ethics committee at the Universidade Federal do Rio Grande do Sul (68271917.7.0000.5347) and permission was granted by the specialized center where data were collected. All patients signed the Free and Informed Consent Form. Instruments were administered by the researcher and interviews were audio recorded and transcribed.

## Results

### Study population

The sample comprised 60 patients who sought or were referred for care and were diagnosed with PTSD. Participants were mostly women, with mean age of 39.05 (SD = 14.41) years. The majority reported sexual violence (n = 20; 33.3%) or the tragic death of an affectively close family member (n = 20; 33.3%) as the index traumatic event (ITE) for seeking care, which in most cases was more than 2 years after the ITE. However, experience of traumatic events prior to the one that motivated the demand for care was also mentioned, with an average of 1.3 (SD = 0.76) events. Table 1 shows the patients’ sociodemographic and clinical characteristics.

**Table 1:** Sociodemographic and clinical characteristics of patients with PTSD

Category/subcategory		n (%)
Sex		
	Female	52 (86.7)
	Male	8 (13.3)
Age (years)		
	18 to 30	20 (33.3)
	31 to 40	11 (18.3)
	41 to 50	12 (20.0)
	51 to 60	17 (28.3)
Ethnicity		
	White	46 (76.7)
	Not white	14 (23.3)
Schooling		
	Illiterate	3 (5.0)
	Elementary school	43 (71.7)
	High school	13 (21.7)
	Higher education	1 (1.7)
Marital status		
	Single	28 (46.7)
	Married/stable relationship	30 (50.0)
	Divorced	3 (3.3)
Income*		
	No income	31 (51.7)
	1 or 2 times the minimum wage	26 (43.3)
	3 times the minimum wage or more	3 (5.0)
ITE		
	Physical violence	4 (6.7)
	Sexual violence	20 (33.3)
	Emotional violence	7 (11.1)
	Urban violence	9 (15.0)
	Tragic death of family member	20 (33.3)
Time elapsed before seeking health care		
	Up to 6 months	13 (24.5)
	From 6 months to 2 years	11 (18.3)
	From 2 to 5 years	9 (15.0)
	From 5 to 10 years	16 (26.7)
Number of traumatic events during life		
	None	8 (13.3)
	1	29 (48.3)
	2	20 (33.3)
	3	3 (5.0)
Past psychiatric problems		
	Yes	48 (80.0)
	No	12 (20.0)
SA (after ITE)		
	Yes	25 (41.7)
	No	35 (58.3)

ITE = index traumatic event; PTSD = post-traumatic stress disorder; SA = suicide attempts.

Results expressed as absolute frequencies (percentages).

* The minimum (monthly) wage was the equivalent of US$186.

### Psychodynamic functioning according to OPD-2

The multiaxial OPD-2 evaluation identifies how patients with PTSD organize their psychodynamic functioning. Axis I evaluates symptoms, their severity, and the subjective suffering of patients with the disease. Table 2 shows the results for the Global Assessment of Functioning (GAF) scale, which, according to the OPD task force,^10^ is a continuum that extends from mental health to disease. The lower the value on a scale from 1 to 100 points, the greater the disease severity. Patients with PTSD recorded a mental, social, and work functioning score of 54.91 points, demonstrating significant symptoms or impairment.

**Table 2:** Axis I - Mean and intensity of items assessed in disease experience and prerequisites for treatment

		Mean (SD)	Intensity			
			1	2	3	4
Objective assessment of the disease/problem						
	GAF (last 7 days)	54.91 (8.31)	-	-	-	-
	EQ-5D	7.16 (1.27)	-	-	-	-
Experience and form of presentation of the disease						
	Subjective suffering	2.49 (0.72)	3 (5)	29 (48.3)	23 (38.3)	0.0

EQ-5D = EuroQol 5 Dimensions; GAF = Global Severity Index; SD = standard deviation.

Results expressed as absolute frequencies (percentages).

The higher the score, the greater the severity. Zero scores are not recorded in the table.

The average score on the EuroQol 5 Dimensions (EQ-5D), which describe the patient's health status on a scale from 1 to 15 points, with severity increasing as scores increase, was 7.16, revealing problems with carrying out daily activities, pain or discomfort, and anxiety or depressive symptoms. The subjective suffering experienced by patients was considered moderate (48.3%), indicating, in addition to the severity of symptoms and their diagnosis, their attitude towards treatment and the way they perceive social contact.^10^

On Axis II, the most representative dysfunctional themes were: 1) experience themselves as isolated and distant in relationships (n = 31; 51.66%), but alert, protecting themselves from attacks (14; 23.33%) due to difficulties in establishing limits for the performance of the other (n = 13; 21.66%); 2) perceive others as negligent and feel abandoned (n = 23; 38.33%) or imposing themselves in a rude way (n = 22; 36.66%), attacking and harming them (n = 14; 23.33%); 3) others experience patients as distant and isolated (n = 28; 46.66%), insufficiently protecting themselves (n = 16; 26.66%) and allowing much space for their performance (n = 14; 23.33%); 4) others experience themselves in the relationship with patients in an ambivalent, distant, and isolated way (n =2 8; 46.66) or excessively caring for and protecting themselves (n = 22; 36.66%), avoiding aggressiveness (n = 14; 23.33%).

**Figure 1:**
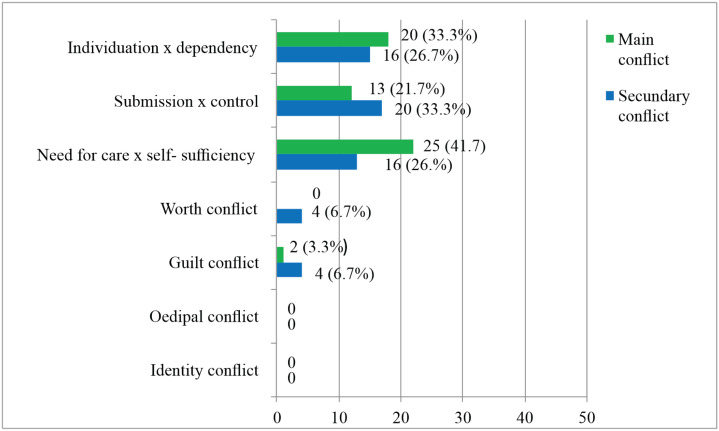
Axis III. Frequency of presentation of conflicts. Results expressed as absolute frequencies (percentages).

On Axis III (Figure 1) the main psychic conflict, which represents a continuum defined from their personal histories, tensions, individual patterns of experience, and behavior,^10^ was "Need for care versus self-sufficiency" (n = 22; 41.5%). In this model, patients are dependent, needing constant proof of others’ attention and care. Their fears relate to being too close or to the fear of losing the other. All conflicts are described in Figure 1.

The way in which the patient manifests his/her conflicts is identified in the guiding, main affect, as mobilized in his/her contact with the relationship network, his/her family, friendships, professional life, social environment, money, body/sexuality and manifestation of diseases. In addition, the behavior observed in contact with the patient in the session is also noted when reporting such interactions. This processing mode can vary among passive/submissive, mixed but passive, mixed but active, and predominantly active modes.^10^ In these patients, all modes of presentation of conflicts were scored, observing that the main mode was the mixed but active mode (n = 20; 33.4%). Thus, patients seek to prove their self-sufficiency in relationships; however, latent depressive feelings emerge as a defense against the feeling of emptiness. Other patients manifested the mixed but passive mode (n = 17; 28.3%), as they appear attached, dependent, or demanding. The need for security is totally denied in the predominantly active mode (n = 14; 23.3%) and separation from the other proves to be impossible in the predominantly passive mode (n = 9; 15%), with impulses to approach and control.

On Axis IV, total self structure was evaluated, which represents the vulnerability and/or availability of the personality's mental functions and the ability to process internal and external stresses and to regulate the self and its relationships.^10^ A moderate/medium level was found (Table 3) (M = 2.15 ± 0.30) and the main anxiety was loss or separation from the object, in addition to fear of one's own internal impulses. In the vast majority of patients, all dimensions of the structural assessment reveal moderate to low intensity level with difficulties in cognitive, emotional, and attachment abilities.

**Table 3:** Axis IV - Mean scores and level of integration of structural personality functions

		Intensity					
		Mean(SD)	1 elevated	1.5	2 moderate	2.5	3 Low
Cognitive abilities							
	Self-perception	2.16 (0.30)	0	3 (5.0)	35 (58.3)	21 (35.0)	1 (1.7)
	Object perception	2.18 (0.29)	0	2 (3.3)	35 (58.3)	22 (36.7)	1 (1.7)
Regulation							
	Self-regulation	2.22 (0.28)	0	1 (1.7)	32 (53.3)	26 (43.3)	1 (1.7)
	Regulation of object relationship	2.24 (0.32)	0	2 (3.3)	30 (50.0)	25 (41.7)	3 (5.0)
Emotional communication							
	Internal communication	2.24 (0.35)	1 (1.7)	3 (5.0)	23 (38.3)	32 (53.3)	1 (1.7)
	Communication with the external world	2.19 (0.33)	1 (1.9)	2 (3.8)	31 (51.7)	25(41.7)	1 (1.7)
Attachment							
	Attachment to internal objects	2.19 (0.29)	0	2 (3.3)	34 (56.7)	23 (38.3)	1 (1.7)
	Attachment to external objects	2.15 (0.34)	1 (1.7)	2 (3.3)	38 (63.3)	16 (26.7)	3 (5.0)
Total structure		2.15 (0.30)	1 (1.7)	2 (3.3)	35 (58.3)	22 (36.7)	0

Results expressed as absolute frequencies (percentages). The closer to 1, the greater the structural integration and the closer to 4, the greater the disintegration. Scores 3.5 and 4 were not endorsed.

On axis V, 41 (68.33) patients had some mental disorder associated with PTSD according to the DSM-5. Diagnoses were made according to DSM-5^1^ criteria after clinical interviews. It was observed that 29 (70.7%) patients had another mental disorder, including major depressive disorder (MDD) (n = 18; 43.9%), borderline disorder (BD) (n = 3; 7.3%), and GAD (n = 8; 19.5%). Moreover, 12 patients (29.3%) had two other associated diagnoses: MDD and GAD (n = 8; 19.5%) and BD and GAD (n = 4; 9.7%).

### Defensive styles according to the DSQ-40

**Figure 2:**
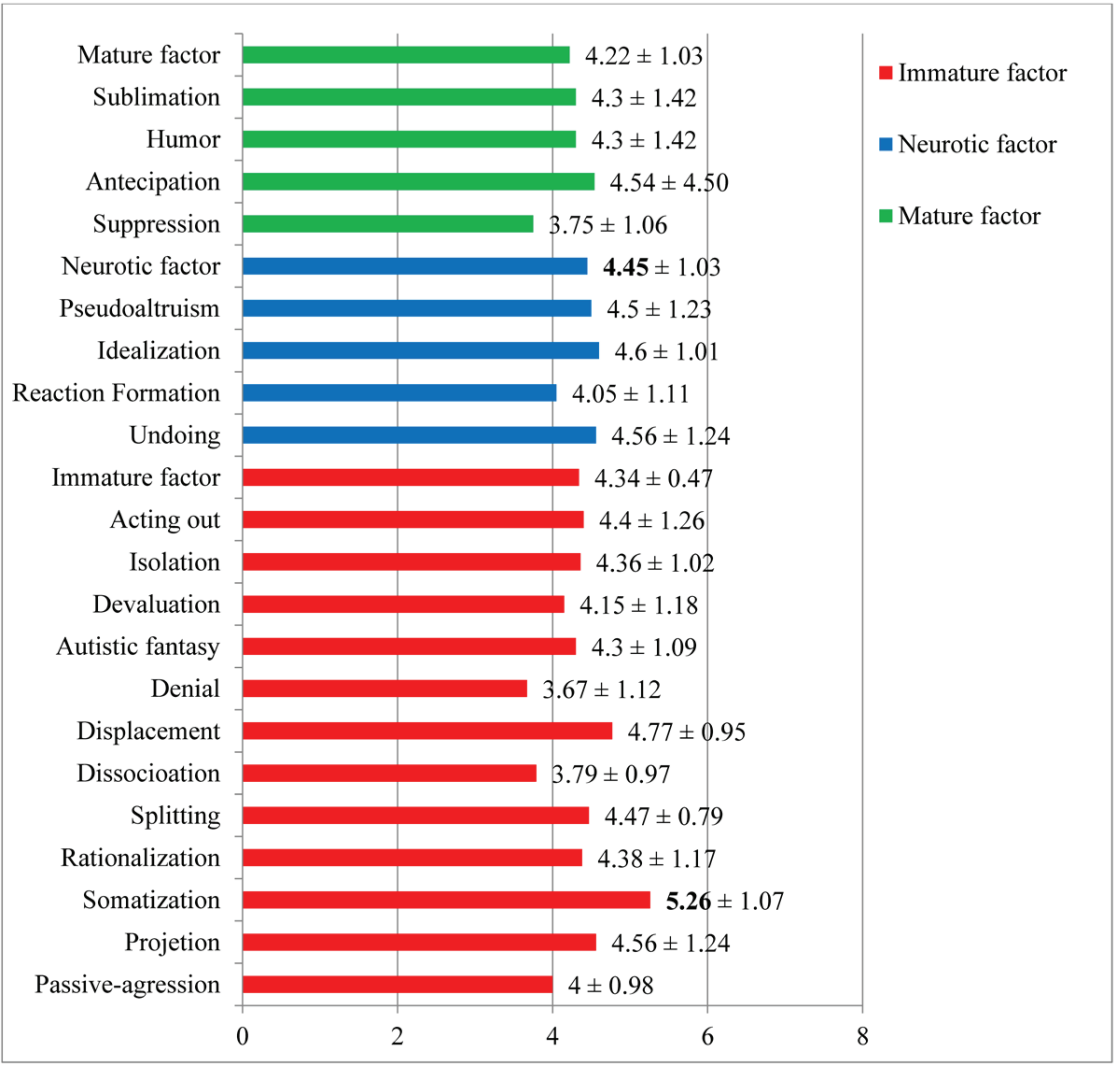
Description of use of defense mechanisms and defensive styles.

Patients with PTSD endorsed the use of all defensive factors, with the neurotic factor being the most prevalent (4.45 ± 0.73). From this perspective, undesirable content comes to consciousness in a disguised or distorted way in order to ward off threatening desires, anxieties, and emotions.^28^ The most used DM was somatization (5.26 ± 1.07), which belongs to the immature factor (5.56 ± 0.98), with a trend to react to stress through somatic manifestations such as pain and bodily but not psychic sensations. Complete results are shown in Figure 2.

Results expressed as mean and standard deviation. The higher the score, the greater the use of defensive mechanisms (DM). The most used DM are in bold font.

### Bivariate analysis

Considering OPD-2, DSQ-40 variables and sociodemographic data, important issues for understanding patients with PTSD were revealed. Axis I: women had better GAF scores (GAF = median_women_ = 55[35-75]; p = 0.015), despite showing symptoms and moderate difficulties in mental, social, and occupational areas. Men, on the other hand, had more significant impairments (GAF_men_ = 50[40-55]).

Furthermore, better GAF scores were identified in patients who had not experienced any other traumatic event prior to the ITE (ITE_none_ = 60[50-70]; p = 0.037), had no previous psychiatric problems (Previous psychiatric problems_none_ = 60[45-70]; p = 0.037), and had not attempted suicide (SA_no_ = 55[45-70]; p = 0.008). Likewise, better quality of life was observed in subjects without psychiatric problems before the ITE (previous psychiatric problems_none_ = 6[5-9]; p = 0.021) and who had not attempted suicide (SA_no_ = 7[5-10]; p = 0.032).

Assessing Axis I of the OPD-2 and DSQ-40, those who rarely presented subjective suffering made greater use of the mature defensive style (subjective suffering_none/rarely_ = 5.88[5.13-5.88]; p = 0.040), of anticipation DM (subjective suffering_none/rarely_ = 5.50[5.50-6.50]; p = 0.032), and of the neurotic defensive style (subjective suffering_none/rarely_ = 5.50[5.13-6.00]; p = 0.046). However, the dissociation DM (previous psychiatric problems_none_ = 4.25; min-max = [3-5.50]; p = 0.005) and rationalization DM (previous psychiatric problems_none_ = 5[4-6]; p = 0.042) were evident in patients who had no psychiatric problems prior to the ITE.

On Axis II, the way in which others report experiencing themselves in relation to the patient as isolated, distant, and withdrawing from relationships was significant in relation to patients who showed low structural level related to attachment to external objects (n = 3 [100%]; p = 0.040). In addition, when investigating the patterns of dysfunctional relationships regarding DM, it was found that patients who reported experiencing themselves as trying not to isolate from relationships more often used the immature defensive style (4.5[2.7-5.7]; p = 0.025) and the autistic fantasy MD (5[2.5-6.0]; p = 0.041). Likewise, in an attempt to stay closer to the patient, the others presented the immature factor (4.5[2.7-5.7]; p = 0.016), the autistic fantasy DM (5[2.5-6.0]; p = 0.01), and rationalization as prevalent (5[2.5-7.0]; p = 0.003) and also the neurotic factor (4.6[3.0-6.1]; p = 0.022). On the other hand, those who experienced themselves in relation to the patient as seeking more contact exhibited greater use of the immature defensive style (4.5[2.7-5.7]; p < 0.001), denial DM (4.0[1.5-6.0]; p = 0.002), cleavage (5[1.5-6.0]; p = 0.025), neurotic style (4.6[3-6.3]; p = 0.014), annulment DM (5.0[2.5-6.5]; p = 0.032), and idealization DM (4.5[3.0-7.5]; p = 0.047).

In relation to psychic conflicts, on Axis III, the "Individuation versus dependence" conflict was identified in patients who had no income (no income_absent/not significant_ = 3 [9.7%]; n_significant/very significant_ = 28 [90.3%]; p = 0.042) or an income of one or two times the minimum wage (= n_absent/not significant_ = 9 [34.6%]; n_significant/very significant_ = 17 [65.4%]; p = 0.042). This conflict was also identified in patients who had experienced trauma during their development (one event = n_absent/not significant_ = 3 [10.3%]; n_significant/very significant_ = 26 [89.7%]; p = 0.042). Moreover, when "Individuation versus dependence" and "Submission versus control" conflicts were more significant, there was greater use of the devaluation DM (Individuation versus dependence_absent/not significant_ = 3[2-6]; Individuation versus dependence_significant/very significant_ = 4.50[2- 8]; p = 0.022; Submission versus control_absent/not significant_ = 3.5[2-5]; and Submission versus control_significant/very significant_ = 4.5[2-8]; p = 0.019). On the other hand, the Self-esteem conflict was more significant in relation to those who used the autistic fantasy DM (Self-esteem_absent/not significant_ = 4.5[1.5-6]; Self-esteem_significant/very significant_ = 5.5[4-6]; p = 0.041).

The mode of functioning of the main conflict was significant in relation to the number of lifetime traumas patients had suffered: those who had suffered trauma before the ITE presented predominantly passive mode (n = 8 [27.6%]; p = 0.021); and patients who had suffered two prior traumas exhibited mixed but active mode (n = 11 [55%]; p = 0.021). This mode of functioning was also observed in those who had attempted suicide (SA_yes_ = 8 [32%]; p = 0.005). On the other hand, patients who did not attempt suicide showed mixed but passive mode of functioning (SA_no_ = 14 [40%]). In addition, there was a predominantly active mode of functioning of the main conflict against the devaluation DM (4.5[2-6]; p = 0.030), autistic fantasy (4.5[3-5.5]; p = 0.034) and dissociation (4[2.5-6.5]; p = 0.043).

When considering the level of structural integration of personality (Axis IV), low attachment to internal objects (>3 times minimum wage = n = 1 [33.3%]; p = 0.29) and low capacity in object perception (>3 times minimum wage = n = 1 [33.3%]; p = 0.29) were found in subjects with income of more than three times the minimum wage. It is noteworthy that patients who reported not having experienced trauma during their development presented high levels of attachment to internal objects (no trauma= n = 2 [25%]; p = 0.032). Likewise, a high level of object regulation was identified in patients with greater use of the displacement mechanism (4.25[4-4.5]; p = 0.028).

## Discussion

This study evaluated the psychodynamic functioning of patients with PTSD relating them to defensive styles, DM, and sociodemographic characteristics. The main findings were: 1) sexual violence was the most common type of trauma; but patients had also suffered other traumas during their lives; 2) In the bivariate analyses, the following was observed: a) Patients who had not suffered traumas during their lives maintained their more organized personality structure (Axis IV), with ability to control impulses and regulate affections, had better GAF and EQ-5D (Axis I), did not attempt suicide and used the displacement mechanism; b) Patients who rarely presented subjective suffering (Axis I) used more flexible defensive styles such as neurotic and mature (anticipation); c) The relationship theme "allowing contact" (Axis II) was significant in relation to use of defenses of the immature style (autistic fantasy, rationalization, denial, cleavage) and neurotic style (annulment and idealization), both in relation to the experiential perspective of the patient and to the others; d) more primitive conflicts (Axis III) were associated with subjects with lower income, who had experienced trauma before the ITE, were more dependent, devalued (Axis II), and used immature defenses (devaluation and autistic fantasy); e) The mode of functioning of the conflict (Axis III) varied in relation to the number of traumas during life, SA, and DM of the immature style (devaluation, autistic fantasy and dissociation).

When evaluating the psychodynamic functioning of patients with PTSD, it was possible to observe, in addition to the current problem, ITE, and symptoms, how the stress caused by the trauma is related to the subjects’ psychic constitution. In general, it is known that trauma, felt as a threat to physical or psychological integrity, produces accumulation of excitations, which are often intolerable to the psychic apparatus.^29^ When producing imbalance, the traumatic experience is relived in order to dominate excitatory stimuli.^30^ Thus, failure to be able to speak about, reflect on, and understand the traumatic event leads to discharge into the body itself, converting affect into a symptom and seeking to repress the anguish aroused by the trauma, as well as its infantile, conscious, or unconscious representations.^31^ The search for relief from tension through repetition of the painful experience and formation of symptoms aims to reduce all the tension of the psychic apparatus, causing it to return to an inanimate state. Freud developed his ideas about the death instinct based on these observations.^32,33^ In the patients evaluated herein, individual characteristics (sex, no other trauma prior to the ITE, personality structure, conflictual schemes, and use of DM) and social factors (interpersonal relationships) influenced modulation of the stress response and the ability to withstand and reorganize themselves in the face of stressful situations.

Consistent with the literature, the majority of patients who developed PTSD were female^1^ with low schooling and low socioeconomic status.^1,29^ In addition, there was wide variation in terms of age among patients, which may be related to the long period of time elapsed between the occurrence of ITE and the search for specialized care, usually over 2 years.

Although no significant associations were found between the ITE and the other variables under study, a high prevalence of sexual violence was observed in the traumatic events that triggered symptoms. This type of aggression is one of the main causes of development of PTSD^34^ in both sexes,^35^ causing feelings of hopelessness, abandonment, and isolation in relation to relationships, increasing the risk of SA.^36^ Such symptoms were present on Axes I, II, and III of the OPD-2 in this study.

In the same proportion, the tragic death of a family member with affective proximity was also revealed as a significant trauma. Unexpected death of family members due to violence or traffic accidents, as well as expected death due to a terminal illness, were associated with the occurrence of PTSD in women with less schooling.^37^ Also, parents who lost children who were victims of violent death presented PTSD between 4 and 60 months after the event,^38^ which indicates the relevance of a specialized perspective on this type of trauma.

In addition to the ITE, many patients revealed that they had experienced trauma prior to the event that caused PTSD, which supports several prior studies.^2,19,39^ Trauma can become devastating, especially in childhood, as object representations become unstable, making it difficult to organize a sense of identity and the ability to reflect/mentalize.^40^ The absence or fragility of the object's response to accept, interpret, and satisfactorily respond to traumatic experiences, combined with the individual's responses to the situation experienced can lead to feelings of helplessness and abandonment, reactivated in other stressful situations.^30^ Thus, exposure to a new trauma or multiple traumas throughout life, and development of PTSD symptoms together with other psychiatric disorders, were shown to be associated with worse global functioning (Axis I), with loss of income, relationship difficulties, feelings of abandonment, and isolation (Axis II), causing a high rate of SA.

Jun et al.^16^ indicated that the less subjects are adapted to reality, the more immature their defenses and the lower their trauma management capacity. Baie et al.^11^ confirmed the relationship between lower level of personality functioning and greater severity of PTSD symptoms. This research extends those results by showing that protection in the face of traumatic experiences enabled formation of a more organized personality structure (Axis IV), constituted by internalization of secure representations, with stable self-structuring. This can be observed in the significant association between patients who reported not having experienced trauma during their development and the high level of attachment to internal objects.

In addition, greater ability to distance oneself, control impulses, and regulate affections and relationships with others, leads to skills of self-perception and perception of others separately, facilitating exchanges with the external world. This makes it possible to trigger DM capable of minimizing anguish by shifting it to less threatening objects (displacement DM). Such characteristics served as a protective factor when experiencing the ITE, allowing these patients to maintain their social, relational, and work functioning, without suicide attempts, even with development of PTSD, unlike patients with more disorganized psychic structure, i.e., with moderate, moderate to low, or low structural levels.

The structural conditions of personality, from organized functioning to severe limitation, interact directly with the manifestation of psychic conflicts and, consequently, the interpersonal relationships established. It was observed that dysfunctional conflicts presented a repetitive pattern in interpersonal and intrapsychic dynamics and aroused affects. The main conflict (Axis III) (Figure 2) identified was the "Need for care versus self-sufficiency," which refers to the desire for security and care in close relationships in the face of dependence and inner emptiness. Those with trauma prior to the ITE and lower income felt even greater helplessness, devaluation, and dependence on the other. Such characteristics corroborate the findings referring to failures in differentiation between the other and the self and, consequently, in the organization of a personality structure with less integration in these patients. "Submission versus control"^10^ was identified as the secondary conflict, in which subjects seek to dominate or submit to others.

The mode of functioning of the main and secondary conflicts, mixed but active modes, points to an excessive attachment to and demand on people with whom they relate. Loss of power is felt as a threat, as negligence, with occurrence of withdrawal. Such characteristics confirm and strengthen findings observed by Both et al.^41^ and Favaretto et al.^19^ in studies on trauma patients.

It is interesting to observe that the greater the number of traumatic events experienced over the course of life, the more primitive and active the conflict (Axis III). Thus, impulse regulation occurred through inflexible defenses, fantasies, or dissociation between trauma and emotion, characteristic of greater structural personality disintegration (Axis IV) and of not being effective. With the intensification of the fear of fusion with the other, these patients isolate themselves even more, increasing their anguish and the need for release, with the passage to the act, the suicide attempt.^10^

The study also investigated use of DM from different factors (immature, neurotic and mature), aiming at protecting the ego faced with a traumatic experience, corroborating other studies on the subject.^14,42,43^ The DM most used was somatization (Figure 2), in which psychic derivatives are converted into bodily symptoms, changing the focus of pain. There was also use of rational explanations for their actions and symptoms, trying to separate the current trauma from consciousness. However, other patients with more flexible defensive style, such as mature and neurotic, were able to anticipate emotions, tolerating anxiety and experiencing the current trauma with less subjective suffering (Axis I).

Isolation DM was evaluated by Jun et al.^16^ and was found to be positively correlated with the severity of PTSD symptoms. In this study, its use stands out in relation to the established dysfunctional patterns of relationships (Axis II), since when patients feel abandoned, perceiving others as negligent or imposing, they isolate themselves. Even those who seek to maintain their relationships end up using immature and neurotic defensive styles, with rational explanations, through which reduction of stress occurs in an imaginative way, without the real approximation of the other.

It is interesting to note that when evaluating how others place themselves in the relationship with patients (Axis II), their defenses are also at a more rigid level, idealizing qualities of themselves and others, denying aspects of reality, trying to repair their actions, or even to separate the good and the bad to preserve their conscience. This characteristic concerns projective identification, being a defense or part of interpersonal communication between patients with PTSD and their relational contacts. It could thus be inferred that patients impose a subtle pressure on others to acquire aspects of their self or a painful internal object in order to dominate and control the other, or even, in order to seize their emotional capacities, controlling their anguish. When the projected material somehow resonates with aspects of the object that pre-existed the projection, the object starts to think, feel, and behave according to what was projected.^44^

Thus, this research provides important evidence on the topic investigated. As limitations, its cross-sectional design and the small number of patients stand out. The variables analyzed could not explain worse global functioning in patients or the relationship between greater personality disruption and higher income. Patients had high symptom intensity and significant difficulties in global functioning. The frequencies of structural variables were concentrated in a single category, and it was not therefore possible to perform analysis through regression, since there was no convergence of results. Bivariate analysis was therefore employed, but a larger sample is needed. In addition, patients who did develop the disease and those who did not manifest it after a traumatic event should also be analyzed. A longitudinal survey could point to the persistence of symptoms and post-traumatic growth related to the findings of this research.

It could be concluded that the OPD-2 was able to assess the psychodynamic functioning characteristics of patients who suffered trauma and developed PTSD, establishing possible factors of protection and vulnerability that explain the lower severity of symptoms and limitations, transgenerational violence, and flaws in the psychic constitution, making the reflexive and defensive process difficult. It was also able to relate to the DSQ-40, complementing understanding of these patients and their defenses.

The possibility of a multiaxial assessment that complements nosological diagnosis helps health professionals to direct psychological/psychiatric treatment, guiding the patient to actively face the disease. The characteristics identified point to a need for work aimed not only at the current moment and the symptoms presented, but also focusing on limitations of the self, building or presenting new elements of secure attachment, offering new connections, and providing the opportunity to differentiate from the other, thus establishing greater stability and structural integration. This reconfiguration becomes possible through the therapist's listening, welcoming, containing, naming, and behavior,^10^ in addition to use of psychotropic drugs when necessary. There is also a need to expand specialized health services for early assessment and intervention with a view to preventing the disorder from becoming chronic. In general, public health policies must direct actions to break the cycle of transgenerational violence and the trivialization of child violence, which are factors of vulnerability when faced with trauma.
