# Supplementary material for: Relationship between psychodynamic functioning, defensive mechanisms, and trauma in patients with post-traumatic stress disorder (PTSD)
Source: Trends Psychiatry Psychother. 2024 Nov 26;46:e20220546. doi: 10.47626/2237-6089-2022-0546 (PMC11790130; doi:10.47626/2237-6089-2022-0546)
Supplement: Supplementary file 1 [file 2238-0019-trends-46-e20220546-suppl01.pdf]

http://doi.org/10.47626/2237-6089-2022-0546

Supplementary Table S1 - Relational themes and items

**Patient experiences himself (through others and with others) often as...**	**Relational themes**	**Patient experiences others often as...**
1. Allowing plenty of space, letting others act autonomously	Allowing space	1. Allowing plenty of space, letting others act autonomously
2. Guiding little, avoiding influence	Guiding others	2. Guiding little, avoiding influence
3. Admiring, idealizing	Recognizing others	3. Admiring, idealizing
4. Apologizing, avoiding censorship	Assigning responsibility	4. Apologizing, avoiding censorship
5. Being overly affectionate	Expressing affection	5. Being overly affectionate
6. Harmonizing, avoiding aggressiveness	Manifest aggressiveness	6. Harmonizing, avoiding aggressiveness
7. Caring much, worrying	Caring	7. Caring much, worrying
8. Imposing yourself rudely	Make contact	8. Imposing yourself rudely
9. Restricting space, intervening	Allowing space	9. Restricting space, intervening
10. Controlling, making claims and demands	Guiding little	10. Controlling, making claims and demands
11. Belittling, devaluing and shaming others	Recognizing others	11. Belittling, devaluing and shaming others
12. Accusing and censoring	Assigning responsibility	12. Accusing and censoring
13. Withdrawing affection	Express affection	13. Withdrawing affection
14. Attacking and harming	Manifest aggressiveness	14. Attacking and harming
15. Neglecting and abandoning	Caring	15. Neglecting and abandoning
16. Pretending, ignoring	Make contact	16. Pretending, ignoring
17. Claiming space and independence	Claim your space	17. Claiming space and independence
18. Challenging and Giving up	Adapting	18. Challenging and Giving up
19. Showing off, making yourself the center of attention	Being assertive	19. Showing off, making yourself the center of attention
20. Denying guilt	Admitting guilt	20. Denying guilt
21. Not reacting when others show affection	Accepting affection	21. Not reacting when others show affection

22. Insufficiently protecting yourself, allowing dangerous developments	Protecting yourself	22. Insufficiently protecting yourself, allowing dangerous developments
23. Depending heavily on others, clinging	Depending on others	23. Depending heavily on others, clinging
24. Having few boundaries, being overly involved	Allowing contact	24. Having few boundaries, being overly involved
25. Avoiding autonomy, seeking guidance	Claiming space	25. Avoiding autonomy, seeking guidance
26. Complaining, abstaining, resigning	Adapting	26. Complaining, abstaining, resigning
27. Belittling yourself, devaluing yourself	Depending on others	27. Belittling yourself, devaluing yourself
28. Blaming yourself	Allowing contact	28. Blaming yourself
29. Closing yourself, running away from other people's affections	Accepting affection	29. Closing yourself, running away from other people's affections
30. Protecting yourself, especially from attacks, being on the alert	Protecting yourself	30. Protecting yourself, especially from attacks, being on the alert
31. Not depending on others, being self-confident	Depending on others	31. Not depending on others, being self-confident
32. Isolating, separating, withdrawing	Allowing contact	32. Isolating, separating, withdrawing

OPD Task Force. Diagnóstico psicodinâmico operacionalizado: manual de diagnostico e plano de tratamento (OPD-2). São Paulo: Hogrefe; 2016. p. 175.
